# Epidemiological patterns of asbestos exposure and spatial clusters of incident cases of malignant mesothelioma from the Italian national registry

**DOI:** 10.1186/s12885-015-1301-2

**Published:** 2015-04-15

**Authors:** Marisa Corfiati, Alberto Scarselli, Alessandra Binazzi, Davide Di Marzio, Marina Verardo, Dario Mirabelli, Valerio Gennaro, Carolina Mensi, Gert Schallemberg, Enzo Merler, Corrado Negro, Antonio Romanelli, Elisabetta Chellini, Stefano Silvestri, Mario Cocchioni, Cristiana Pascucci, Fabrizio Stracci, Elisa Romeo, Luana Trafficante, Italo Angelillo, Simona Menegozzo, Marina Musti, Domenica Cavone, Gabriella Cauzillo, Federico Tallarigo, Rosario Tumino, Massimo Melis, Sergio Iavicoli, Alessandro Marinaccio, ReNaM Working Group

**Affiliations:** 1 Epidemiology Unit, Department of Occupational and Environmental Medicine, Epidemiology and Hygiene, Italian Workers’ Compensation Authority (INAIL), Rome, Italy; 2 Regional Operating Center of Valle d’Aosta (COR Valle d’Aosta), Valle d’Aosta Health Local Unit, Aosta, Italy; 3 COR Piedmont, Unit of Cancer Prevention, University of Turin and CPO-Piemonte, Torino, Italy; 4 COR Liguria, Epidemiology and Prevention Department, National Cancer Research Institute (IST), Genova, Italy; 5 COR Lombardy, Department of Preventive Medicine, Fondazione IRCCS Ca’ Granda, Ospedale Maggiore Policlinico and University of Milan, Milano, Italy; 6 COR Province of Trento, Provincial Unit of Health, Hygiene and Occupational Medicine, Trento, Italy; 7 COR Veneto, Occupational Health Unit, Department of Prevention, Padua, Italy; 8 COR Friuli-Venezia Giulia, University of Trieste -Trieste General Hospitals, Clinical Unit of Occupational Medicine, Trieste, Italy; 9 COR Emilia-Romagna, Health Local Unit, Public Health Department, Reggio Emilia, Italy; 10 COR Tuscany, Cancer Prevention and Research Institute, Unit of Environmental and Occupational Epidemiology, Firenze, Italy; 11 COR Marche, Environmental and Health Sciences Department, University of Camerino, Hygienistic, Camerino, Italy; 12 COR Umbria, University of Perugia, Department of Hygiene and public health, Perugia, Italy; 13 COR Lazio, Department of Experimental Medicine, University La Sapienza, Roma, Italy; 14 COR Abruzzo, Health Local Unit, Occupational Medicine Unit, Pescara, Italy; 15 COR Campania, Department of Experimental Medicine, Second University of Naples, Napoli, Italy; 16 COR Puglia, Department of Internal Medicine and Public Medicine, University of Bari, Section of Occupational Medicine “B. Ramazzini”, Bari, Italy; 17 COR Basilicata, Epidemiologic Regional Center, Potenza, Italy; 18 COR Calabria, Public Health Unit, Crotone, Italy; 19 COR Sicily, “Civile - M.P. Arezzo” Hospital, Ragusa Cancer Register Unit, Ragusa, Italy; 20 COR Sardegna, Regional Epidemiological Center, Cagliari, Italy

**Keywords:** Mesothelioma, Asbestos, National registry, Clusters, Italy

## Abstract

**Background:**

Previous ecological spatial studies of malignant mesothelioma cases, mostly based on mortality data, lack reliable data on individual exposure to asbestos, thus failing to assess the contribution of different occupational and environmental sources in the determination of risk excess in specific areas. This study aims to identify territorial clusters of malignant mesothelioma through a Bayesian spatial analysis and to characterize them by the integrated use of asbestos exposure information retrieved from the Italian national mesothelioma registry (ReNaM).

**Methods:**

In the period 1993 to 2008, 15,322 incident cases of all-site malignant mesothelioma were recorded and 11,852 occupational, residential and familial histories were obtained by individual interviews. Observed cases were assigned to the municipality of residence at the time of diagnosis and compared to those expected based on the age-specific rates of the respective geographical area. A spatial cluster analysis was performed for each area applying a Bayesian hierarchical model. Information about modalities and economic sectors of asbestos exposure was analyzed for each cluster.

**Results:**

Thirty-two clusters of malignant mesothelioma were identified and characterized using the exposure data. Asbestos cement manufacturing industries and shipbuilding and repair facilities represented the main sources of asbestos exposure, but a major contribution to asbestos exposure was also provided by sectors with no direct use of asbestos, such as non-asbestos textile industries, metal engineering and construction. A high proportion of cases with environmental exposure was found in clusters where asbestos cement plants were located or a natural source of asbestos (or asbestos-like) fibers was identifiable. Differences in type and sources of exposure can also explain the varying percentage of cases occurring in women among clusters.

**Conclusions:**

Our study demonstrates shared exposure patterns in territorial clusters of malignant mesothelioma due to single or multiple industrial sources, with major implications for public health policies, health surveillance, compensation procedures and site remediation programs.

**Electronic supplementary material:**

The online version of this article (doi:10.1186/s12885-015-1301-2) contains supplementary material, which is available to authorized users.

## Background

Malignant mesothelioma (MM) is an uncommon and high mortality neoplasm typically originating in mesothelial cells lining the body’s serous cavities, mainly the pleura and the peritoneum. The risk of MM attributable to asbestos exposure has been reported to be between 86% and 95% in most recent epidemiological studies [1-3]. The rate of non-asbestos-related MM varies widely among studies and is likely influenced by the different methods of assessing exposure [4].

From the 1950’s until the total national ban in 1992, Italy was an important producer and user of asbestos and asbestos-containing materials. In particular, asbestos production reached a peak in the 1976–1980 period, but remained steadily over 100,000 tons/year until 1987. Moreover, asbestos imports still exceeded 50,000 tons/years in 1991. These temporal patterns made the peak in asbestos consumption later in Italy than in other European countries and in the United States [5]. Therefore, considering the long latency of MM (generally around 35–40 years from first exposure), a high number of cases is still expected in Italy in the next few decades [6].

In Italy a national registry of malignant mesothelioma cases (ReNaM) was instituted by law in 2002 implementing previous regional surveillance experiences and adopting centrally defined procedures and methods in performing active case ascertainment and asbestos exposure assessment. Cases are collected through regionally operational units. Death schedules and hospital discharge records are used to check the incidence data completeness. Standardized guidelines are applied to definition of cases of MM and to evaluation of both occupational and environmental exposure to asbestos, which is performed by direct interviews with MM sufferers or their close relatives.

The geographic distribution of MM cases can reflect either the past use of asbestos at local facilities or particular situations of environmental contamination from both natural and anthropic sources. Several studies have shown the usefulness of identifying territorial clusters of MM for public health policies in Italy [7,8] and elsewhere [9,10]. Using Bayesian methods looks very promising for estimating the MM risk at the level of small territorial units and for more effectively detecting clusters [10,11]. However, ecological spatial studies are almost always affected by bias due to the lack of individual exposure data, thus failing to provide any evidence of causation [12,13]. This also made it difficult to evaluate the contribution of occupational and environmental sources of asbestos exposure in the determination of risk excess in specific areas. This information may be useful for refining predictions and more accurately drawing up remediation plans.

This study aims to identify territorial clusters of MM cases in Italy through a Bayesian spatial analysis and to characterize them for exposure patterns by integrated use of individual asbestos exposure information retrieved from the Italian national mesothelioma registry (ReNaM).

## Methods

The ReNaM periodically acquires data from the regional operational units, as required by law, treating them anonymously through proper encrypting procedures in order to ensure privacy. Aggregated data are made publicly available through periodical reports [14] and a set of fixed tables is freely downloadable in the open data section of the site of the institution. The registry currently holds data about cases of malignant mesothelioma occurring between 1993 and 2008 referring to subjects resident in Italy. Data from cases with 2009–2013 diagnoses are under acquisition and subject to completeness analysis at this moment. The registry covers almost all the national territory except for one region (Molise) and one autonomous province (Bolzano) where regionally operational units have not been activated yet. Nevertheless, the collection of incidence data is still partial in two other regions (Calabria and Sardinia). Since no experimental procedures were conducted on study participants, ethical approval was not required under national legislation. Only cases occurring in regions and during periods with complete incidence data were extracted for analysis from the national database. In detail, data were available for 16 out of 20 Italian regions and an autonomous province, corresponding to 7,063 (out of 8,101) municipalities overall and to almost 92% of the total Italian population as computed in the national 2001 and 2011 census. Incident malignant mesothelioma cases were assigned to the municipality of residence at the time of diagnosis. Person-years were obtained by summing the resident population, reconstructed yearly by the National Institute of Statistics (ISTAT), during the incidence period for each municipality. Standardized incidence ratios were used to compare observed cases to those expected based on the age-specific rates of the Italian geographic area where each municipality was located (Northwest, Northeast, Centre, South and Islands).

A geographical cluster analysis was performed for each area applying a Bayesian hierarchical model [15]. A conditional auto-regressive (CAR) effect was used to account for the spatial structure of the municipal-level data that effectively “borrows” information from adjacent municipalities to improve estimates for individual municipalities. This approach reduces the variance in the associated estimates and allows for the spatial effect of regional differences in risk exposure. Relative risk (RR) estimates were derived from a posterior sampling approach and expressed as means.

The formulation of the model is:

$$ {\mathrm{O}}_{\mathrm{i}} \sim \mathrm{Poisson}\left({\mathrm{E}}_{\mathrm{i}}{\uplambda}_{\mathrm{i}}\right) $$

$$ \log \left({\uplambda}_{\mathrm{i}}\right) = \upalpha + {\mathrm{U}}_{\mathrm{i}} + {\mathrm{V}}_i $$

where λ_i_ is the relative risk in the i*th* municipality, O_i_ is the number of observed mesothelioma cases, E_i_ are the expected cases, U_i_ and V_i_ are the unstructured and structured random effects, which were assigned a normal and a conditional autoregressive prior distribution, respectively and α is the overall risk level assuming the effects of U_i_ and V_i_ equal to zero. Unstructured heterogeneity variance and conditional spatial variance were each given gamma prior distributions with scale parameter 0.5 and shape parameter 0.005.

The model was implemented using the GeoBUGS package included in the WinBUGS software package, freely distributed by the WinBUGS Project at Cambridge University [16]. WinBUGS software uses the Markov chain Monte Carlo method to fit the statistical model. Two chains and 20,000 iterations were run, so the results were based on 40,000 samples. Geographical data were mapped using MapInfo software v. 8 (Pitney Bowes, Inc., Troy, N.Y.).

After mapping the RR estimates, territorial MM clusters were defined based on the following criteria: a group of neighboring municipalities (a) all showing RR > 1 and (b) including one or more municipalities with a 90% credibility interval for RR entirely above 1.

The second step of this study consisted in characterizing the identified geographical clusters by using data about asbestos exposure from the ReNaM at the municipal level. The information available for analysis includes occupational, familial and residential histories collected by specifically trained health professionals through standardized interviews with MM sufferers or their closest relatives if deceased. Informed consent is routinely obtained from the interviewed subjects or their surviving relatives. Exposure was classified by regional occupational health experts using likelihood criteria into the following categories: occupational, further specified as definite, probable or possible; familial (i.e. cohabitation with one or more asbestos workers); environmental (living in proximity to industrial facilities directly using asbestos or asbestos containing material or in areas with naturally occurring asbestos or asbestos-like ore in the soil); other non-occupational (indirect use of asbestos containing products at home or during leisure time); unlikely or unknown, as more extensively described elsewhere [17]. In detail, among residents in every municipality included in the clusters, occupational and familial cases were described by the industrial activities to which asbestos exposure had been attributed by occupational health physicians or experts. Definite, probable and possible cases of occupational origin were taken together for analysis in order to improve detection of putative industrial sources in individual territorial settings. No direct data about the municipality of the working facilities of the MM cases or their relatives were available in the central database. Nevertheless, the presence of this kind of occupational sources was verified within each cluster by sharing the analysis findings with regional operational units. In the absence of occupational or familial exposure, environmental cases were assessed in relation to exposure to natural or industrial sources, mainly asbestos product manufacturing plants, based on the information collected from the referents of regional operational units and from the scientific literature. The attribution of cases to residential exposure was made at the regional level taking into account both the distance from the environmental source and the period of residence as indicated in the standardized questionnaire [17], but no rigid criteria of definition were fixed. Whenever an occupational, familial or environmental MM case was attributable to more than one industrial activity it was computed repeatedly for each source identified.

## Results

A total of 15,322 incident cases of all-site malignant mesothelioma were analyzed from the ReNaM, accounting for 96.7% (15,322/15,845) of all cases recorded in the 1993–2008 period. The main characteristics of the MM cases are described by gender in Table 1. Most MM cases (93%) occurred in the pleural site. The male/female ratio was 2.5 overall but clearly lower for pericardial (1.9) and peritoneal sites (1.4). The mean age at diagnosis was 69.3 years with no significant difference by gender (70.3 for women and 68.9 for men). Less than 10% of cases occurred in subjects younger than 55 years. About 78% of cases showed a definite diagnosis of malignant mesothelioma, i.e. one confirmed through immunohistochemical and/or histological examination, with a higher percentage in men than in women.

**Table 1:** **Incident cases of malignant mesothelioma recorded by the Italian national mesothelioma registry (ReNaM) selected for the cluster analysis**

		Men		Women	
		Number of cases	%	Number of cases	%
Age class	0-54	1,046	9.6	450	10.3
	55-64	2,646	24.1	856	19.7
	65-74	4,015	36.6	1,404	32.2
	75+	3,257	29.7	1,648	37.8
Period of diagnosis	1993-1996	1,042	9.5	382	8.8
	1997-2000	2,271	20.7	884	20.3
	2001-2004	3,706	33.8	1,503	34.5
	2005-2008	3,945	36.0	1,589	36.4
Area	Northwest	5,158	47.1	2,423	55.6
	Northeast	2,586	23.6	901	20.7
	Centre	1,450	13.2	454	10.4
	South & Islands	1,770	16.1	580	13.3
Diagnostic certainty	Definite	8,708	79.4	3,234	74.2
	Probable	1,142	10.4	574	13.2
	Possible	1,114	10.2	550	12.6
Site of lesion	Pleura	10,312	94.0	3,941	90.4
	Peritoneum	579	5.3	403	9.3
	Pericardium	26	0.2	14	0.3
	Testis	47	0.4	-	-
Exposure ascertainment	Direct interview	4,585	41.8	1,401	32.2
	Indirect interview	4,139	37.8	1,727	39.6
	No interview	2,240	20.4	1,230	28.2
Type of exposure	Occupational, definite	4,987	45.5	526	12.1
	Occupational, probable	890	8.1	112	2.6
	Occupational, possible	1,291	11.8	402	9.2
	Familial	76	0.7	446	10.2
	Environmental	214	1.9	294	6.7
	Other non-occupational	80	0.7	108	2.5
	Unlikely	193	1.8	223	5.1
	Unknown	993	9.1	1,017	23.3
	Undefined	2,240	20.4	1,230	28.2
Total		10,964		4,358	

Italy, 1993–2008 (N = 15,322).

Exposure was defined by direct or indirect interview for 11,852 out of 15,322 cases (77.3%) with a large variability among regions. Among the interviewed subjects exposure to asbestos was ascertained in 86.4% (7,538/8,724) of men and in 60.3% (1,888/3,128) of women. The percentage of unknown or unlikely exposure to asbestos in all the cases taken together varied widely according to whether the interviewee was the MM sufferer (15.4%) or one of his/her relatives (25.6%).

**Figure 1:**
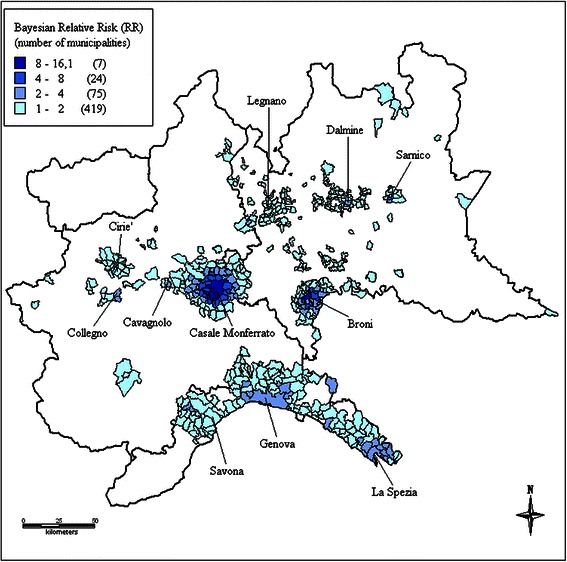
Identified clusters of malignant mesothelioma cases in the Northwest, Italy, ReNaM, 1993–2008. Smoothed relative risk (RR) estimates of incident cases of mesothelioma (all sites) recorded by the Italian registry of malignant mesothelioma (ReNaM) in the 1993–2008 period are mapped based on municipality of residence. Only municipalities with RR higher than 1 are shown in the figure and color-coded. Cluster labels refer to the municipality with the highest number of cases.

**Figure 2:**
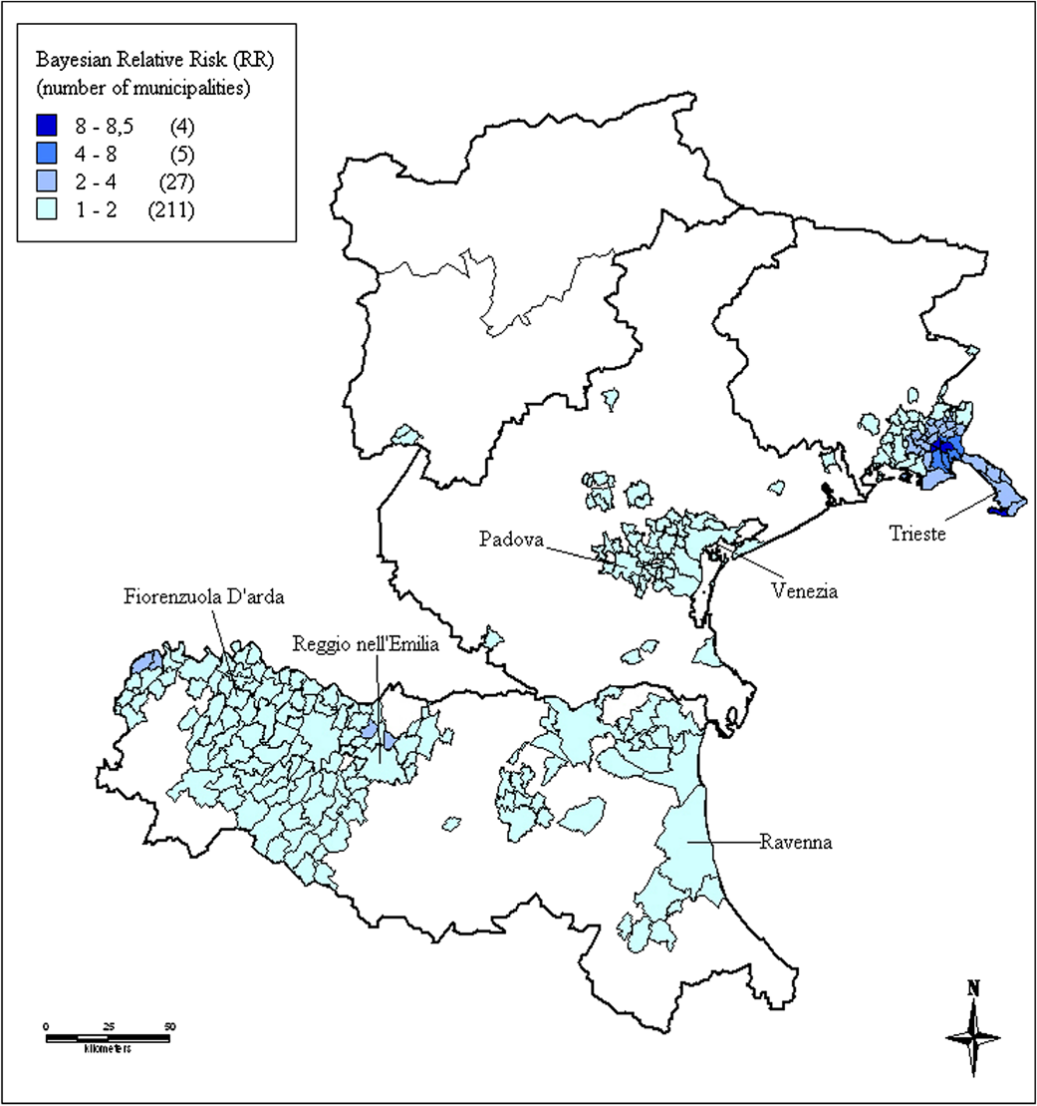
Identified clusters of malignant mesothelioma cases in the Northeast, Italy, ReNaM, 1993–2008. Smoothed relative risk (RR) estimates of incident cases of mesothelioma (all sites) recorded by the Italian registry of malignant mesothelioma (ReNaM) in the 1993–2008 period are mapped based on municipality of residence. No incidence data are available for autonomous province of Bolzano. Only municipalities with RR higher than 1 are shown in the figure and color-coded. Cluster labels refer to the municipality with the highest number of cases.

**Figure 3:**
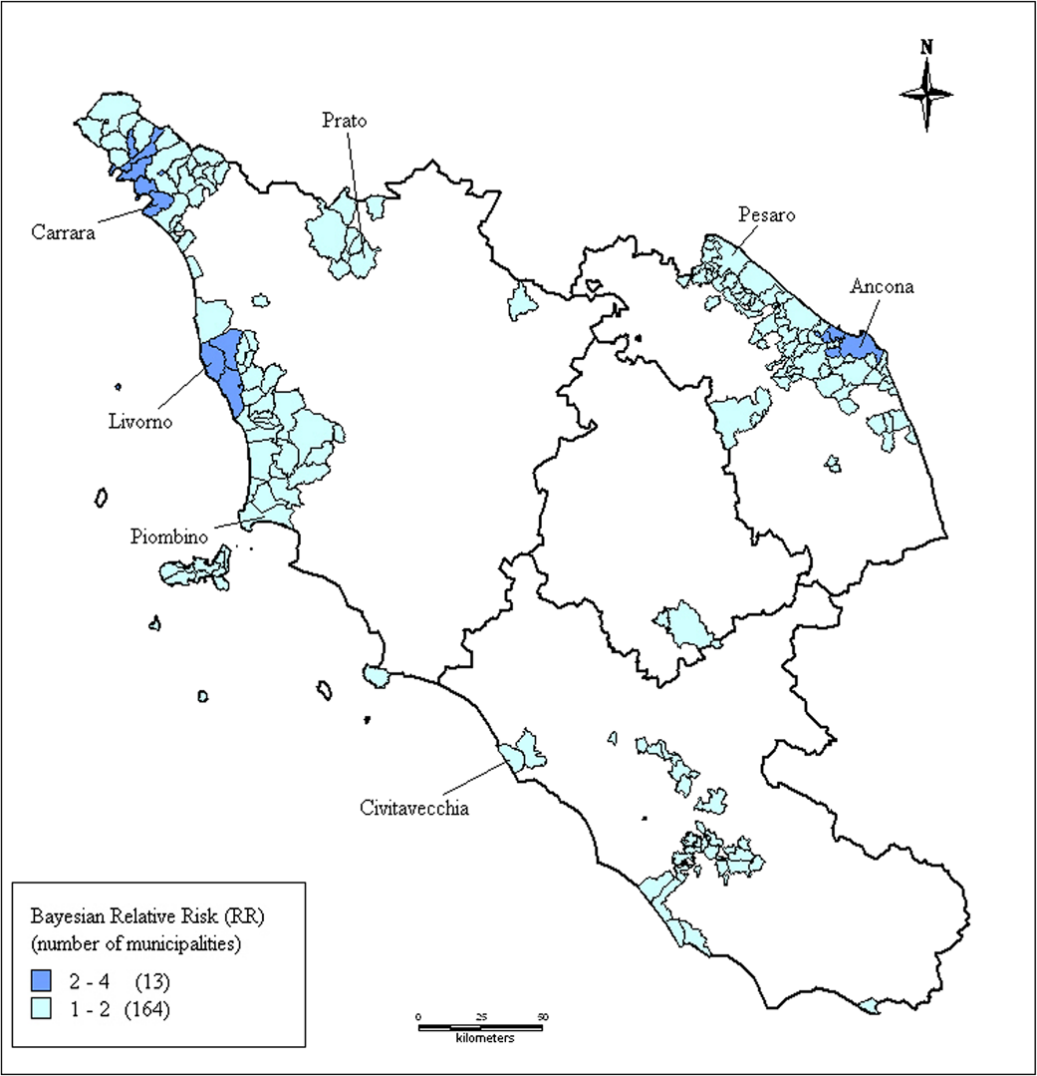
Identified clusters of malignant mesothelioma cases in the Centre, Italy, ReNaM, 1993–2008. Smoothed relative risk (RR) estimates of incident cases of mesothelioma (all sites) recorded by the Italian registry of malignant mesothelioma (ReNaM) in the 1993–2008 period are mapped based on municipality of residence. Only municipalities with RR higher than 1 are shown in the figure and color-coded. Cluster labels refer to the municipality with the highest number of cases.

**Figure 4:**
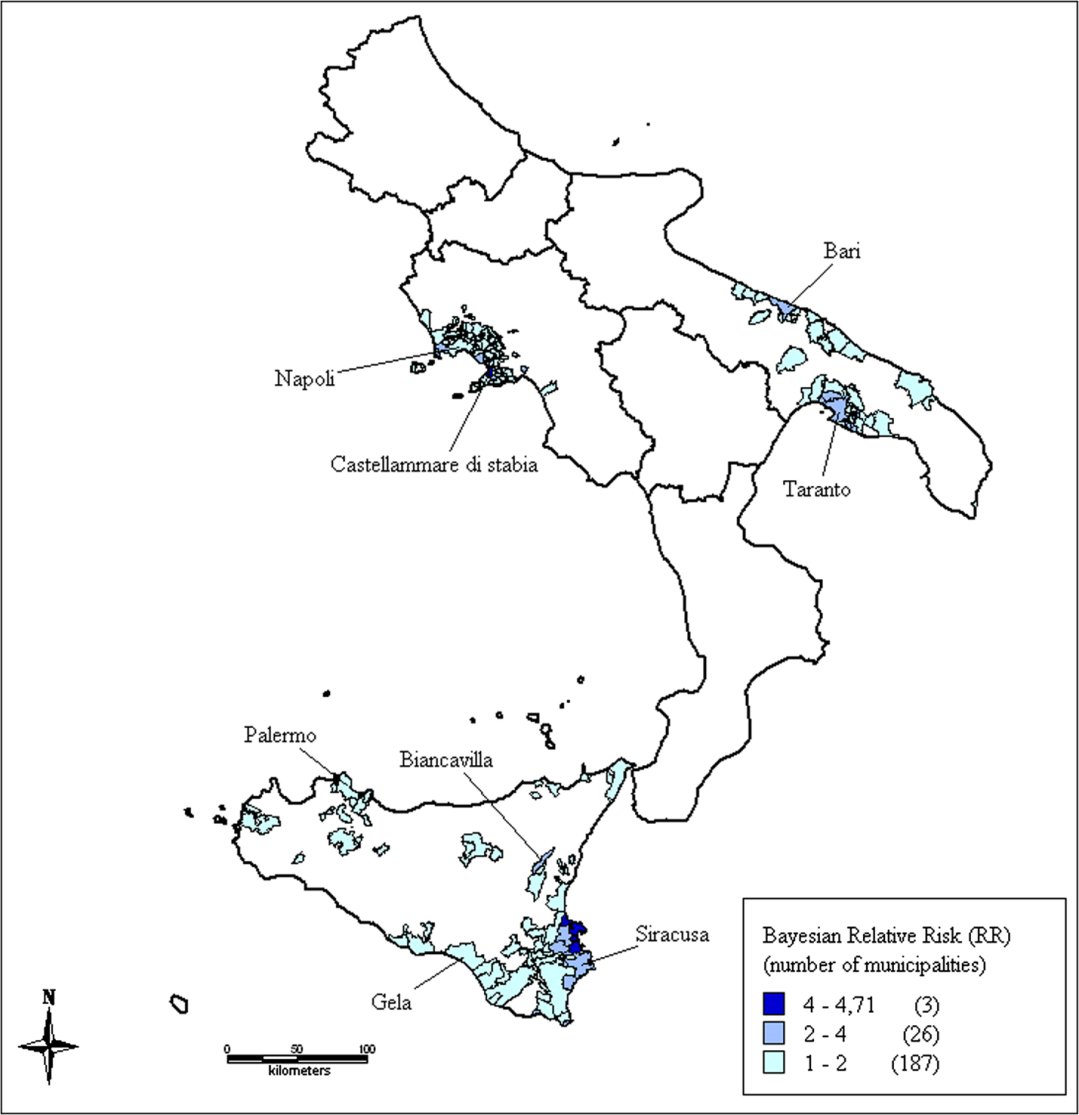
Identified clusters of malignant mesothelioma cases in South & Islands, Italy, ReNaM, 1993–2008. Smoothed relative risk (RR) estimates of incident cases of mesothelioma (all sites) recorded by the Italian registry of malignant mesothelioma (ReNaM) in the 1993–2008 period are mapped based on municipality of residence. No incidence data are available for Molise, Calabria and Sardinia (not shown). Only municipalities with RR higher than 1 are shown in the figure and color-coded. Cluster labels refer to the municipality with the highest number of cases.

In Figures 1, 2, 3 and 4 color-coded maps of unadjusted smoothed RR are presented referring to the four geographic areas of Italy. A total of 32 clusters were identified, mostly located in Northern Italy (11 in the Northwest and 6 in the Northeast) (Table 2). Maps of uncorrected standardized incidence ratios and of distribution of posterior probability of the estimated RR exceeding 1 for all areas are reported in Additional files 1, 2, 3, 4, 5, 6, 7 and 8.

**Table 2:** **Identified clusters of malignant mesothelioma cases by territorial area, number of municipalities included, number of cases (total, female) and modality ofasbestos exposure (defined, environmental and unknown)**

ID	Cluster	Area	Municipalities	MM cases			Asbestos exposure				
				Total	Women		Defined	Environmental		Unknown	
			No.	No.	No.	%	No.	No.	%	No.	%
1	Casale Monferrato	Northwest	89	703	293	41.7	508	109	21.5	10	2.0
2	Cavagnolo	Northwest	10	40	11	27.5	21	1	4.8	1	4.8
3	Ciriè	Northwest	25	95	37	38.9	67	8	11.9	0	-
4	Collegno	Northwest	10	219	94	42.9	136	11	8.1	1	0.7
5	Dalmine	Northwest	70	246	84	34.1	246	2	0.8	54	21.9
6	Genoa	Northwest	65	1,246	243	19.5	1,092	6	0.5	180	16.5
7	Legnano	Northwest	62	350	114	32.6	344	5	1.4	61	17.7
8	Sarnico	Northwest	12	44	23	52.3	44	0	-	4	9.1
9	Savona	Northwest	35	170	34	20.0	148	3	2.0	28	18.9
10	Broni	Northwest/ Northeast	78	256	107	41.8	253	43	17.0	46	18.2
11	La Spezia	Northwest/Centre/ Northeast	69	456	59	12.9	440	6	1.4	39	8.9
12	Fiorenzuola d’Arda	Northeast	41	158	59	37.3	145	5	3.4	20	13.8
13	Padua	Northeast	14	159	58	36.5	151	13	8.6	15	9.9
14	Ravenna	Northeast	20	247	64	25.9	236	5	2.1	23	9.7
15	Reggio nell’Emilia	Northeast	20	162	46	28.4	150	3	2.0	14	9.3
16	Trieste	Northeast	55	625	91	14.6	618	1	0.2	71	11.5
17	Venice	Northeast	19	331	73	22.1	318	12	3.8	25	7.9
18	Carrara	Centre/ Northwest	7	107	14	13.1	106	0	-	15	14.1
19	Ancona	Centre	37	152	30	19.7	124	0	-	15	12.1
20	Civitavecchia	Centre	2	13	0		12	0	-	0	-
21	Leghorn	Centre	23	244	40	16.4	244	1	0.4	26	10.7
22	Pesaro	Centre	21	66	19	28.8	59	1	1.7	11	18.6
23	Piombino	Centre	14	42	9	21.4	42	0	-	4	9.5
24	Prato	Centre	7	115	28	24.3	115	2	1.7	14	12.2
25	Bari	South & Islands	13	258	67	26.1	245	46	18.8	8	3.3
26	Biancavilla	South & Islands	4	29	11	37.9	17	7	41.2	3	17.6
27	Castellammare di Stabia	South & Islands	55	135	36	26.7	60	3	5.0	6	10.0
28	Gela	South & Islands	6	37	4	10.8	29	1	3.4	9	31.0
29	Naples	South & Islands	40	312	72	23.1	108	1	0.9	13	12.0
30	Palermo	South & Islands	13	217	44	20.3	76	1	1.3	22	28.9
31	Syracuse	South & Islands	28	166	33	19.9	70	4	5.7	21	30
32	Taranto	South & Islands	19	212	38	17.9	190	13	6.8	2	1.0

Italy, 1993-2008.

The distribution of cases in the clusters by gender and by geographic area, as well as the environmental fraction of cases (i.e. the percentage of cases attributed to environmental exposure to asbestos based on expert evaluation of the information collected through interviews) and the size of unrecognized exposures among defined cases are shown in Table 2. Table 3 reports detailed information about asbestos exposure referring to the main economic activities involved, i.e. those causing more than 3% of defined MM cases in each cluster. For several large clusters less represented economic sectors (less than 3%) also need to be mentioned. A significant number of MM cases was attributable in the Genoa cluster to military defense (N = 28), an oil refinery (N = 19) and an electric power plant (N = 13), and in the Trieste cluster to many wood furniture factories (N = 14), a steel industry (N = 12) and an oil refinery (N = 11). Whenever available, the most relevant literature references are included for the individual industrial sites in Table 3.

**Table 3:** **Identified clusters of malignant mesothelioma cases by economic sector and modality of asbestos exposure (E = environmental; F = familial; O = occupational)**

ID		Main economic sectors and number of exposures*	Reference
1	Casale Monferrato	Asbestos cement industry: N = 251 (E = 101, F = 59, O = 91); Construction: N = 43 (F = 12, O = 31); Metal engineering: N = 29 (O); Road transportation: N = 27 (F = 1, O = 26)	[19,28]
2	Cavagnolo	Asbestos cement industry: N = 8 (E = 1, F = 7); Construction: N = 3 (O); Electric power plant: N = 3 (O); Automotive industry: N = 2 (O)	
3	Ciriè	Non-asbestos textile industry: N = 11 (F = 1, O = 10); Asbestos textile industry: N = 10 (E = 1, F = 2, O = 7); Mining: N = 6 (E = 3, O = 3); Construction: N = 5 (O); Metal engineering: N = 5 (O); Rubber industry: N = 5 (O)	[44,45]
4	Collegno	Asbestos textile industry: N = 40 (E = 8, F = 4, O = 28); Automotive industry: N = 16 (E = 6, F = 2, O = 8); Metal engineering: N = 14 (F = 1, O = 13); Construction: N = 11 (F = 1, O = 10); Plastics industry: N = 10 (F = 1, O = 9); Food industry: N = 9 (O); Non-asbestos textile industry: N = 8 (F = 1, O = 7); Rubber industry: N = 6 (O)	[24]
5	Dalmine	Metal product manufacturing: N = 41 (O); Non-asbestos textile industry: N = 33 (O); Construction: N = 27 (O); Metal engineering: N = 20 (O); Asbestos cement industry: N = 8 (F = 1, O = 7); Chemical industry: N = 8 (O)	
6	Genoa	Shipyard: N = 269 (F = 17, O = 252); Port (handling and shipping): N = 183 (F = 14, O = 169); Construction: N = 102 (F = 4, O = 98); Steel industry: N = 90 (E = 1, F = 4, O = 85); Metal product manufacturing: N = 81 (F = 1, O = 80); Metal engineering: N = 51 (F = 5, O = 46)	[21]
7	Legnano	Non-asbestos textile industry: N = 88 (F = 1, O = 87); Metal engineering: N = 50 (O); Construction: N = 40 (F = 1, O = 39); Metal product manufacturing: N = 30 (O); Chemical industry: N = 10 (O)	
8	Sarnico	Non-asbestos textile industry: N = 22 (F = 1, O = 21); Asbestos textile industry: N = 7 (O); Construction: N = 3 (O); Rubber industry: N = 2 (O)	[25]
9	Savona	Chemical industry: N = 24 (O); Construction: N = 20 (O); Port (shipping and handling): N = 11 (O); Shipyard: N = 9 (O); Glass industry: N = 7 (O); Metal product manufacturing: N = 7 (O); Manufacture of basic metals: N = 6 (O); Military defense: N = 6 (O); Railway carriage construction and maintenance: N = 5 (O)	[21]
10	Broni	Asbestos cement industry: N = 84 (E = 41, F = 15, O = 28); Construction: N = 31 (O)	[20]
11	La Spezia	Shipyard: N = 233 (E = 2, F = 14, O = 217); Construction: N = 50 (F = 2, O = 48); Port (handling and shipping): N = 41 (O); Military defense: N = 32 (O); Metal engineering: N = 32 (F = 1, O = 31); Oil refinery: N = 21 (O); Metal product manufacturing: N = 18 (O); Electric power plant: N = 14 (O); Manufacture of basic metals: N = 14 (E = 1; F = 2, O = 11)	[21,33]
12	Fiorenzuola d’Arda	Construction: N = 26 (F = 5, O = 21); Food industry: N = 18 (O); Glass industry: N = 11 (E = 4, F = 1, O = 6); Metal engineering: N = 10 (O); Metal product manufacturing: N = 8 (O)	
13	Padua	Railway carriage construction and maintenance: N = 22 (E = 4, F = 1, O = 17); Construction: N = 19 (F = 4, O = 15); Metal engineering: N = 14 (F = 3, O = 11); Asbestos cement industry: N = 8 (E = 4, O = 4); Railway transport: N = 7 (E = 6, O = 1); Sugar industry: N = 6 (F = 2, O = 4); Non-asbestos textile industry: N = 6 (O)	[37]
14	Ravenna	Construction: N = 33 (F = 1, O = 32); Chemical industry: N = 26 (E = 1, F = 2, O = 23); Sugar industry: N = 22 (F = 1, O = 21); Metal engineering: N = 13 (O); Manufacture of synthetic fibers: N = 9 (F = 2, O = 7); Shipyard: N = 8 (E = 1, F = 1, O = 6)	[41]
15	Reggio nell’Emilia	Asbestos cement industry: N = 44 (E = 2, F = 2, O = 40); Railway carriage construction and maintenance: N = 31 (F = 7, O = 24); Construction: N = 18 (F = 1, O = 17); Non-asbestos textile industry: N = 6 (O)	[47,48]
16	Trieste	Shipyard: N = 241 (E = 1, F = 21, O = 219); Construction: N = 74 (F = 1, O = 73); Metal engineering: N = 54 (F = 1, O = 53); Port (handling and shipping): N = 48 (O); Metal product manufacturing: N = 45 (O)	[22,23]
17	Venice	Shipyard: N = 55 (E = 1, F = 9, O = 45); Construction: N = 54 (F = 5, O = 49); Port (handling and shipping): N = 41 (E = 1, F = 4, O = 36); Chemical Industry: N = 37 (E = 2, F = 1, O = 34); Metal engineering: N = 34 (F = 2, O = 32); Metal product manufacturing: N = 18 (O); Manufacture of basic metals: N = 17 (F = 3, O = 14); Glass industry: N = 12 (E = 1, O = 11)	[29]
18	Carrara	Shipyard: N = 25 (O); Construction: N = 25 (O); Metal engineering: N = 18 (O); Metal product manufacturing: N = 14 (F = 1, O = 13); Port (handling and shipping): N = 12 (O); Stone extraction and cutting: N = 10 (O); Motor vehicle repairing: N = 7 (O); Asbestos cement industry: N = 5 (F = 2, O = 3)	[49]
19	Ancona	Shipyard: N = 39 (F = 2, O = 37); Construction: N = 17 (F = 1, O = 16); Military defense: N = 9 (O); Asbestos cement industry: N = 8 (O)	[50]
20	Civitavecchia	Port (handling and shipping): N = 4 (O); Electric power plant: N = 4 (O); Construction: N = 4 (O); Metal engineering: N = 2 (O)	[51]
21	Leghorn	Construction: N = 56 (F = 1, O = 55); Shipyard: N = 48 (F = 2, O = 46); Metal product manufacturing: N = 45 (F = 1, O = 44); Military defense: N = 31 (O); Metal engineering: N = 31 (O); Port (handling and shipping): N = 25 (F = 1, O = 24); Chemical industry: N = 19 (O); Glass industry: N = 18 (O); Electric power plant: N = 16 (F = 3, O = 13); Food industry: N = 16 (O); Agriculture: N = 15 (F = 2, O = 13); Asbestos cement industry: N = 10 (E = 1, O = 9); Motor vehicle repairing: N = 9 (O)	[30,52]
22	Pesaro	Construction: N = 8 (O); Metal engineering: N = 5 (O); Military defense: N = 5 (O)	
23	Piombino	Steel industry: N = 17 (F = 1, O = 16); Construction: N = 12 (O); Port (shipping and handling): N = 8 (O); Military defense: N = 5 (O); Metal product manufacturing: N = 4 (O); Agriculture: N = 4 (O); Food industry: N = 3 (O)	[30]
24	Prato	Non-asbestos textile industry: N = 39 (O); Wholesale of non-metal waste and scraps: N = 30 (O); Railway carriage construction and maintenance: N = 19 (E = 1, F = 1, O = 17); Construction: N = 14 (O); Agriculture: N = 13 (O); Manufacture of wood furniture: N = 10 (O); Metal engineering: N = 7 (O)	[35,38]
25	Bari	Asbestos cement industry: N = 59 (E = 42, F = 3; O = 14); Construction: N = 22 (O); Military defense: N = 21 (O); Railway transport: N = 19 (E = 4, O = 15); Port (handling and shipping): N = 17 (O); Metal product manufacturing: N = 8 (O)	[26]
26	Biancavilla	Mining: N = 7 (E); Construction: N = 3 (O)	[42,43]
27	Castellamare di Stabia	Shipyard: N = 20 (F = 4, O = 16); Asbestos cement industry: N = 6 (E = 1, O = 5); Port (shipping): N = 5 (E = 1, O = 4); Manufacture of basic metals: N = 5 (O); Metal product manufacturing: N = 4 (F = 2, O = 2); Manufacture of jewelry: N = 3 (O)	
28	Gela	Construction: N = 5 (O); Oil refinery: N = 2 (O); Automotive industry: N = 2 (O)	[53]
29	Naples	Asbestos cement industry: N = 17 (F = 1, O = 16); Steel industry: N = 15 (F = 1, O = 14); Railway carriage construction and maintenance: N = 8 (O); Shipyard: N = 6 (O); Motor vehicle repairing: N = 6 (O); Port (handling and shipping): N = 4 (O)	[39,54]
30	Palermo	Shipyard: N = 31 (E = 1, F = 1, O = 29); Railway transport: N = 6 (F = 1, O = 5); Construction: N = 4 (O)	[55]
31	Syracuse	Construction: N = 16 (O); Asbestos cement industry: N = 6 (E = 2, O = 4); Port (shipping): N = 5 (E = 1, O = 4); Shipyard: N = 3 (E = 2, O = 1); Metal engineering: N = 3 (O)	
32	Taranto	Shipyard: N = 54 (E = 4, F = 4, O = 45); Steel industry: N = 45 (E = 2, F = 2, O = 41); Military defense: N = 40 (F = 1, O = 39); Construction: N = 15 (O); Port (handling and shipping): N = 7 (E = 3, O = 4)	[32]

*Only economic sectors accounting for more than 3% of defined cases are reported.

The main sources of asbestos-related mesotheliomas are shown to be asbestos cement manufacturing plants and shipyards. However a significant contribution to asbestos exposure is also provided by sectors with no direct use of asbestos, such as non-asbestos textiles, metal engineering, metal product manufacturing and construction. Cases for which environmental exposure was ascertained are mostly concentrated in clusters where asbestos cement plants were located, but a few situations of external contamination by natural asbestos or asbestos-like fibers are also detected.

Some aggregates of municipalities shown in Figure 1 are not identifiable as clusters based on the criteria applied. This is the case of the area of Molina di Ledro (7 cases of MM in total) in the Northeast, where a factory producing pre-shaped asbestos-containing insulating building materials has been operating since 1973 [18]. This is also the case of two areas in the Northwest. In the first one, Angera-Verbania (52 cases of MM in total), many chemical manufacturing plants were located, including one facility producing a specific asbestos containing insulation material, angerite, widely used in the Italian chemistry sector. In the last, Savigliano-Fossano, a significant number of cases were railway carriage workers. At least one municipality within the area shows significantly high SIR value in the first two cases (Pieve di Ledro: 17.98, 95%CI 3.61-52.53; Molina di Ledro: 8.98, 2.41-22.98; Angera: 3.84, 1.75-7.30), but for Savigliano the SIR 95% confidence interval was not statistically significant (Savigliano: 1.59, 0.99-2.43).

## Discussion

Some critical limitations of the ReNaM dataset have to be discussed first. Some Italian regions, mainly in the South-Islands area, still do not contribute to the incidence data. This incomplete territorial coverage could partially explain the smaller number of clusters detected in Southern Italy. On the other hand the effectiveness of identification of the modalities of exposure is not fully consistent among regions since the percentage of interviewed subjects varies between 45% and 95% depending on available resources and knowledge. The number of MM cases classified as unknown is actually negligible in some clusters but exceeds a fourth of all the defined cases in others, as detailed in Table 2. Furthermore, since the asbestos exposure coding system is completely qualitative, heterogeneity among regions in assigning an exposure code is a real issue, despite the national guidelines. As a result exposure characterization is likely less accurate in a few areas than in others.

Despite these limitations, this study is the first to our knowledge to integrate a cluster analysis of MM incident cases with individual exposure figures, allowing reconstruction of different patterns of exposure to asbestos. The availability of individual data made it possible to overcome the possibly biased approaches using spatial distance from putative environmental sources as a proxy of exposure [13]. On the other hand this study does confirm the correspondence between spatial clustering of mesothelioma cases and either occupational or environmental sources of asbestos exposure as identified case by case through expert-based assessment.

In particular our findings confirm the relevance of the major direct use made of asbestos in Italy both in the manufacture of so-called “Eternit” construction materials and in shipyard insulation activities starting from the first decades of the nineties. The largest MM clusters, per number of cases or municipalities included, were found, indeed, where the biggest asbestos cement plants [19,20] or shipyard facilities [21-23] were located. Several clusters were also attributable to exposure in the asbestos textile industry [24,25]. Overall, it should be noted that an asbestos cement industry, an asbestos textile industry or a harbor industrial area inclusive of shipyards partially contribute to exposure of MM cases in about 75% of the clusters identified.

Clusters in which the primary source of exposure was an asbestos cement industry are also characterized by a high proportion of cases with environmental exposure (above 15% for Casale Monferrato, Bari and Broni) [26,27]. The high number of MM cases occurring in women in the two largest asbestos cement industry-centered clusters (Casale Monferrato and Broni) may actually be due to more common both familial and environmental exposure to asbestos [28]. Conversely, the percent female contribution is clearly lower in other clusters where multiple industrial sources were identified. In the surrounding risk areas near asbestos cement industries a significant number of cases of malignant mesothelioma also occurred due to occupational exposure in the construction sector (such as in the clusters around Casale Monferrato, Broni, Bari, Reggio nell’Emilia, Padua and Syracuse), likely because of the more intensive use of asbestos cement products more easily available at the local level.

Among clusters located in the proximity of harbors, asbestos-related mesotheliomas - mostly of occupational origin -were mainly associated with naval construction and/or repair activities (Genoa and La Spezia in the Northwest, Trieste and Venice in the Northeast, Ancona, Carrara and Leghorn in the Centre, Castellammare di Stabia, Naples, Palermo and Taranto in the South) [21,22,29,30] or shipping and handling of goods possibly including raw asbestos. A number of cases were also seen in first-degree relatives of workers engaged in these activities limited to the biggest clusters (Genoa, La Spezia and Trieste) [23,31]. Occupational exposure is also attributed, to a varying extent, to other industrial activities preferentially concentrated in harbor areas, such as steel manufacturing plants (Genoa, Piombino, Naples-Bagnoli, Taranto and Trieste) [21,30,32], oil refineries (La Spezia, Gela, Genoa and Trieste) [33] chemical facilities (Leghorn, Ravenna, Savona, Venice-Marghera) [21,30] and electric power plants (La Spezia, Leghorn, Civitavecchia, Genoa) [34]. Another circumstance of exposure was found to be working in metal product manufacturing, mostly in the manufacture of structural metal products (11 out of 15 cases in Carrara, 21 out of 62 in Genoa, 16 out of 18 in La Spezia, 34 out of 44 in Leghorn, 33 out of 45 in Trieste, 10 out of 18 in Venice). In all these settings workers could be indirectly exposed while inhaling asbestos fibers spread into the air during maintenance of the insulated machinery or structural frameworks or tanks.

Industrial sectors characterized by indirect use of asbestos have also been found to contribute significantly to MM cases in several clusters, namely the non-asbestos textile industry (see Sarnico, Legnano, Cirié, Prato, Dalmine and Padua) [25,35,36], railway carriage construction and maintenance (clusters of Padua, Reggio nell’Emilia, Naples and Prato) [37-39] and metal engineering (Legnano). The clusters where the non-asbestos textile industry was found to represent a significant source of asbestos occupational exposure (Collegno and Sarnico) also showed a relatively high percentage of cases occurring in women, likely due to the lower gender gap in employment in this particular sector. In Sarnico a unique situation was documented where a non-asbestos textile industry was operating adjacent to an asbestos textile plant that shared the neighboring area [25]. In the cluster of Legnano a number of cases were found to be workers in a factory which made industrial machinery and equipment. This may be due to spreading of fibers from asbestos blankets used in preheating and slow cooling of weld joints of large pipes or tanks and to the asbestos textile protective cloths used. Less common occupational sectors of asbestos exposure were also found to be considerable for incidence of malignant mesothelioma in specific local situations, including the automotive industry (Collegno) and the wholesale trade in non-metal waste and scrap (Prato). In the latter case, asbestos exposure may be related to sorting and reuse of asbestos contaminated jute sacks [40]. Further occupational settings deserving attention in clusters are sugar refineries (Ravenna, Padua) [41], manufacture of wood furniture (Prato, Trieste), glass industry (Fiorenzuola d’Arda, Leghorn, Savona, Venice) and stone extraction and cutting (Carrara).

Finally, in a few clusters a specific pattern of exposure to asbestos was found in relation to the presence of natural sources. Environmental cases actually predominate in the Biancavilla cluster, this being a town in eastern Sicily where exposure was attributed to naturally occurring rocks containing fluoro-edenite fibers (an asbestiform amphibole-like mineral) extracted from a local stone quarry. The epidemiological evidence of an excess of mortality for pleural malignant mesothelioma in this area has actually led to the identification of this previously unknown fiber and of its carcinogenicity by the scientific community [42,43]. In the Cirié cluster, a large number of environmental cases is explained by the proximity to the Balangero asbestos mine, closed in 1990 [44,45].

Taken together our data support the existence of shared exposure patterns in territorial clusters of malignant mesothelioma due to single or multiple industrial sources. Moreover, a large number of cases with ascertained environmental origin were detected in clusters. These findings have important implications both for preventive and compensation purposes. Post-occupational health surveillance of asbestos workers is still a key issue for the Italian regional health care system. Moreover, possible government action is under discussion in Italy for compensation of subjects whose malignant mesothelioma is caused by environmental or household exposure to asbestos, since at the moment reparations for damages can only be obtained through lawsuits.

Starting from a national database, the methodological approach based on Bayesian smoothing techniques allowed us to provide more reliable estimates of cancer risk using small area (municipality) count data and so to identify geographical clusters of incident malignant mesothelioma with high specificity. In this connection, applying proper definition criteria, clusters of very different size and shape were identified but all were confirmed to include significant industrial sources of asbestos exposure [46]. However, this analysis can fail to detect excess risk related to small-sized areas as a consequence of spatial smoothing in a limited number of situations [11]. According to our experience in such instances a concurrent evaluation of uncorrected SIRs, always supported by exposure information, warrants a critical evaluation.

In this study the municipality of residence of MM sufferers at the time of the diagnosis was taken as a proxy for that of exposure. For occupational cases the extent of the possible misclassification is expected to be lower in the cluster analysis with respect to classical municipality-level risk estimation through uncorrected SIRs. Moreover, the availability of precise information about the source of residential exposure made it possible correctly to attribute to each industrial site the environmental cases occurring among residents. The direct definition of municipality of occupational exposure is on going through further collaboration with the operational regional centers. This future step will make it possible to minimize misclassification in geographical attribution of exposure and to improve the accuracy of our findings.

## Conclusions

Based on a large epidemiological surveillance system of incident malignant mesothelioma cases this study is the first to integrate a Bayesian territorial cluster analysis with standardized exposure data collection. This approach was found to provide unique information about country-level patterns of asbestos-related health risks in order to target public health policies and to improve effectiveness of site remediation and health care actions.

## Additional files

Additional file 1: **Distribution of unadjusted standardized incidence ratio (SIR) of malignant mesothelioma in the Northwest, Italy, ReNaM, 1993–2008.** Crude SIRs of malignant mesothelioma (all sites) recorded by the Italian registry of malignant mesothelioma (ReNaM) in the 1993–2008 period are mapped based on municipality of residence.
Additional file 2: **Distribution of unadjusted standardized incidence ratio (SIR) of malignant mesothelioma in the Northeast, Italy, ReNaM, 1993–2008.** Crude SIRs of malignant mesothelioma (all sites) recorded by the Italian registry of malignant mesothelioma (ReNaM) in the 1993–2008 period are mapped based on municipality of residence. No incidence data are available for the autonomous province of Bolzano.
Additional file 3: **Distribution of unadjusted standardized incidence ratio (SIR) of malignant mesothelioma in the Centre, Italy, ReNaM, 1993–2008.** Crude SIRs of malignant mesothelioma (all sites) recorded by the Italian registry of malignant mesothelioma (ReNaM) in the 1993–2008 period are mapped based on municipality of residence.
Additional file 4: **Distribution of unadjusted standardized incidence ratio (SIR) of malignant mesothelioma in South & Islands of Italy, ReNaM, 1993–2008.** Crude SIRs of malignant mesothelioma (all sites) recorded by the Italian registry of malignant mesothelioma (ReNaM) in the 1993–2008 period are mapped based on municipality of residence. No incidence data are available for Molise, Calabria and Sardinia (not shown).
Additional file 5: **Distribution of the posterior probability of smoothed relative risk (RR) being greater than 1 for malignant mesothelioma in the Northwest, Italy, ReNaM, 1993–2008.** The mean posterior probability of RR >1 for malignant mesotheliomas (all sites) recorded by the Italian registry of malignant mesothelioma (ReNaM) in the 1993–2008 period is mapped based on municipality of residence.
Additional file 6: **Distribution of the posterior probability of smoothed relative risk (RR) being greater than 1 for malignant mesothelioma in the Northeast, Italy, ReNaM, 1993–2008.** The mean posterior probability of RR >1 for malignant mesotheliomas (all sites) recorded by the Italian registry of malignant mesothelioma (ReNaM) in the 1993–2008 period is mapped based on municipality of residence. No incidence data are available for the autonomous province of Bolzano.
Additional file 7: **Distribution of the posterior probability of smoothed relative risk (RR) being greater than 1 for malignant mesothelioma in the Centre, Italy, ReNaM, 1993–2008.** The mean posterior probability of RR >1 for malignant mesotheliomas (all sites) recorded by the Italian registry of malignant mesothelioma (ReNaM) in the 1993–2008 period is mapped based on municipality of residence.
Additional file 8: **Distribution of the posterior probability of smoothed relative risk (RR) being greater than 1 for malignant mesothelioma in South & Islands, Italy, ReNaM, 1993–2008.** The mean posterior probability of RR >1 for malignant mesotheliomas (all sites) recorded by the Italian registry of malignant mesothelioma (ReNaM) in the 1993–2008 period is mapped based on municipality of residence. No incidence data are available for Molise, Calabria and Sardinia (not shown).
