# Supplementary figures and images for: Epidemiological patterns of asbestos exposure and spatial clusters of incident cases of malignant mesothelioma from the Italian national registry
Source: BMC Cancer. 2015 Apr 15;15:286. doi: 10.1186/s12885-015-1301-2 (PMC4404011; doi:10.1186/s12885-015-1301-2)

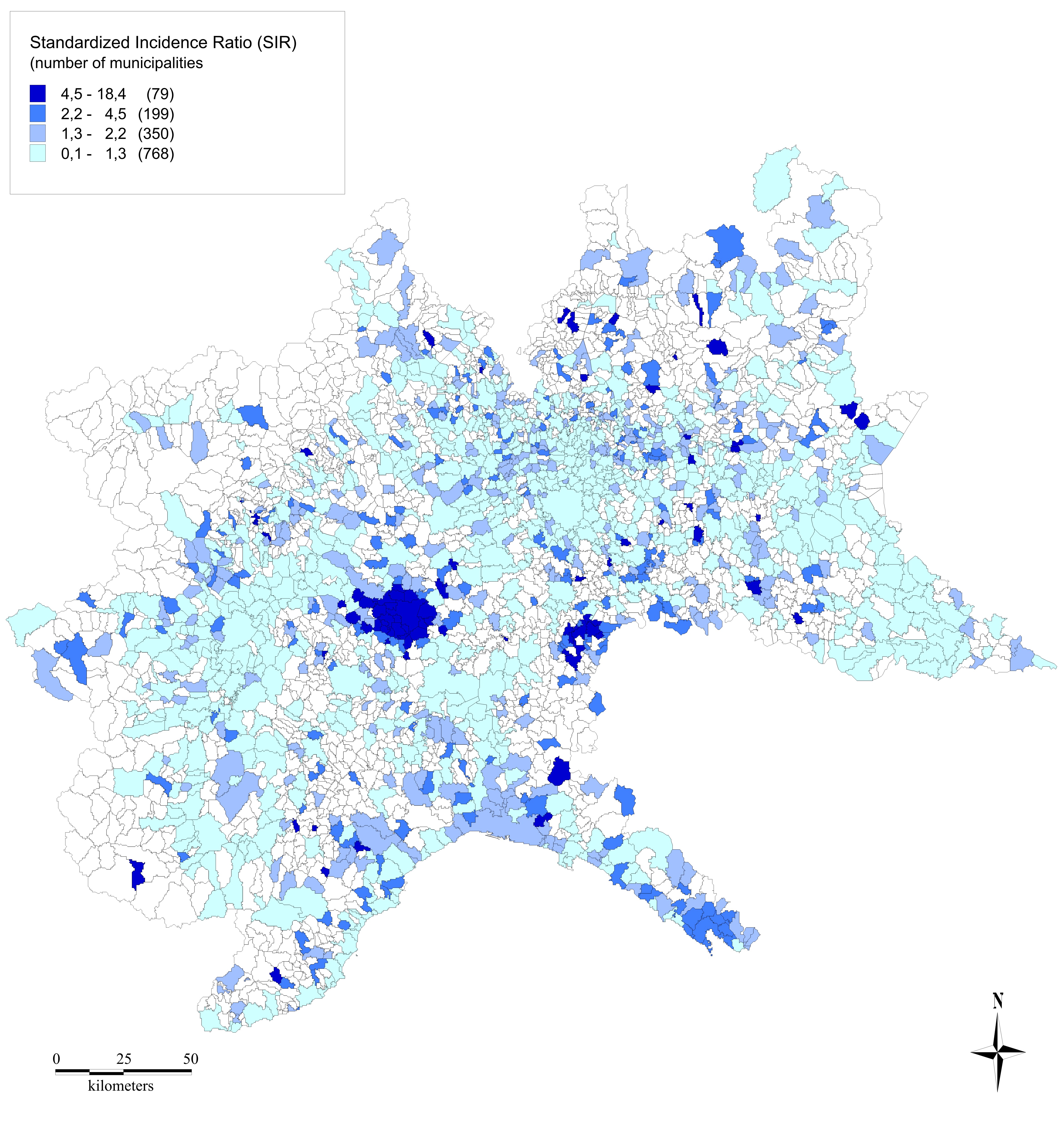

Supplement: Additional file 1: — Distribution of unadjusted standardized incidence ratio (SIR) of malignant mesothelioma in the Northwest, Italy, ReNaM, 1993–2008. Crude SIRs of malignant mesothelioma (all sites) recorded by the Italian registry of malignant mesothelioma (ReNaM) in the 1993–2008 period are mapped based on municipality of residence. [file 12885_2015_1301_MOESM1_ESM.jpeg]

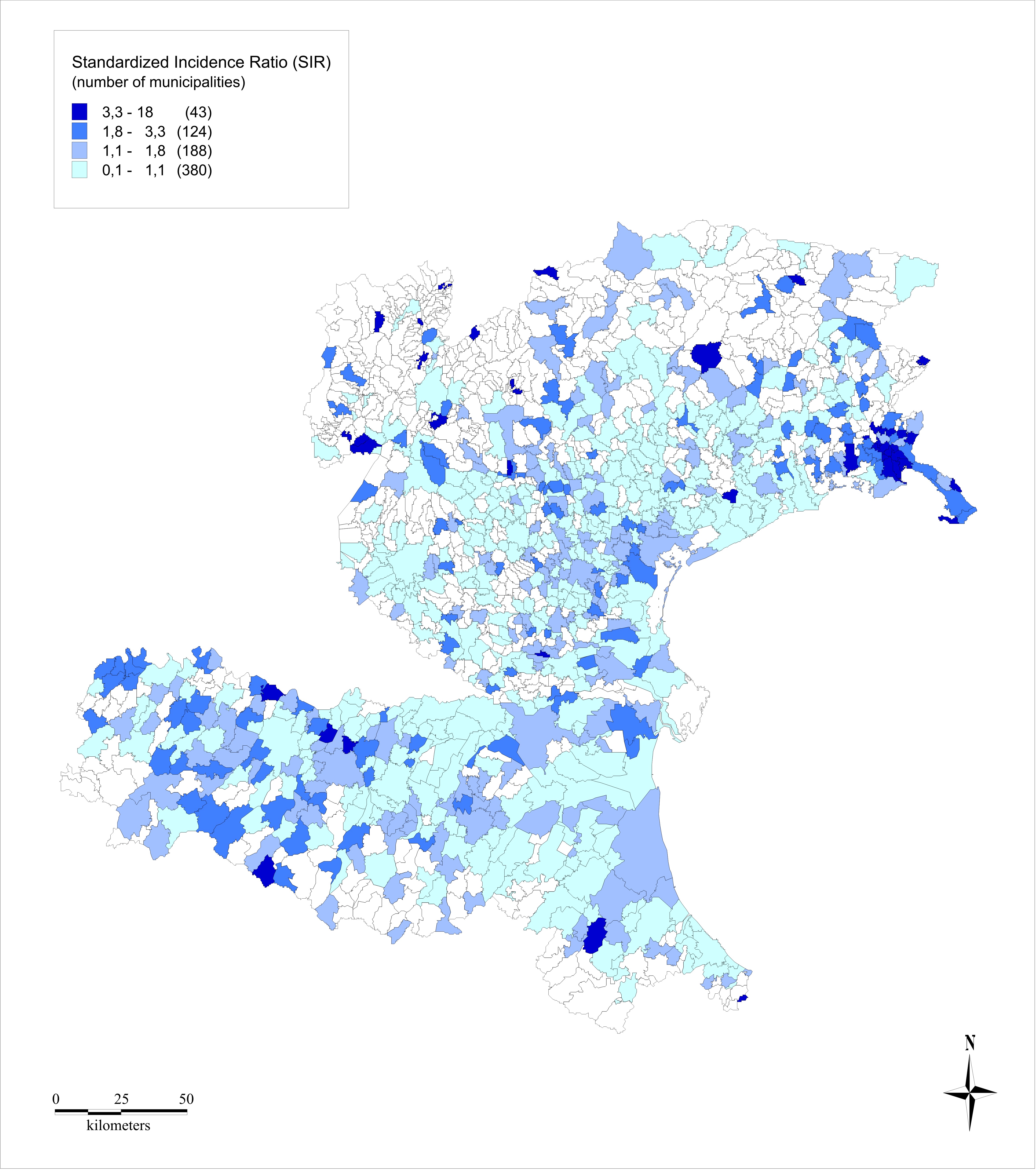

Supplement: Additional file 2: — Distribution of unadjusted standardized incidence ratio (SIR) of malignant mesothelioma in the Northeast, Italy, ReNaM, 1993–2008. Crude SIRs of malignant mesothelioma (all sites) recorded by the Italian registry of malignant mesothelioma (ReNaM) in the 1993–2008 period are mapped based on municipality of residence. No incidence data are available for the autonomous province of Bolzano. [file 12885_2015_1301_MOESM2_ESM.jpeg]

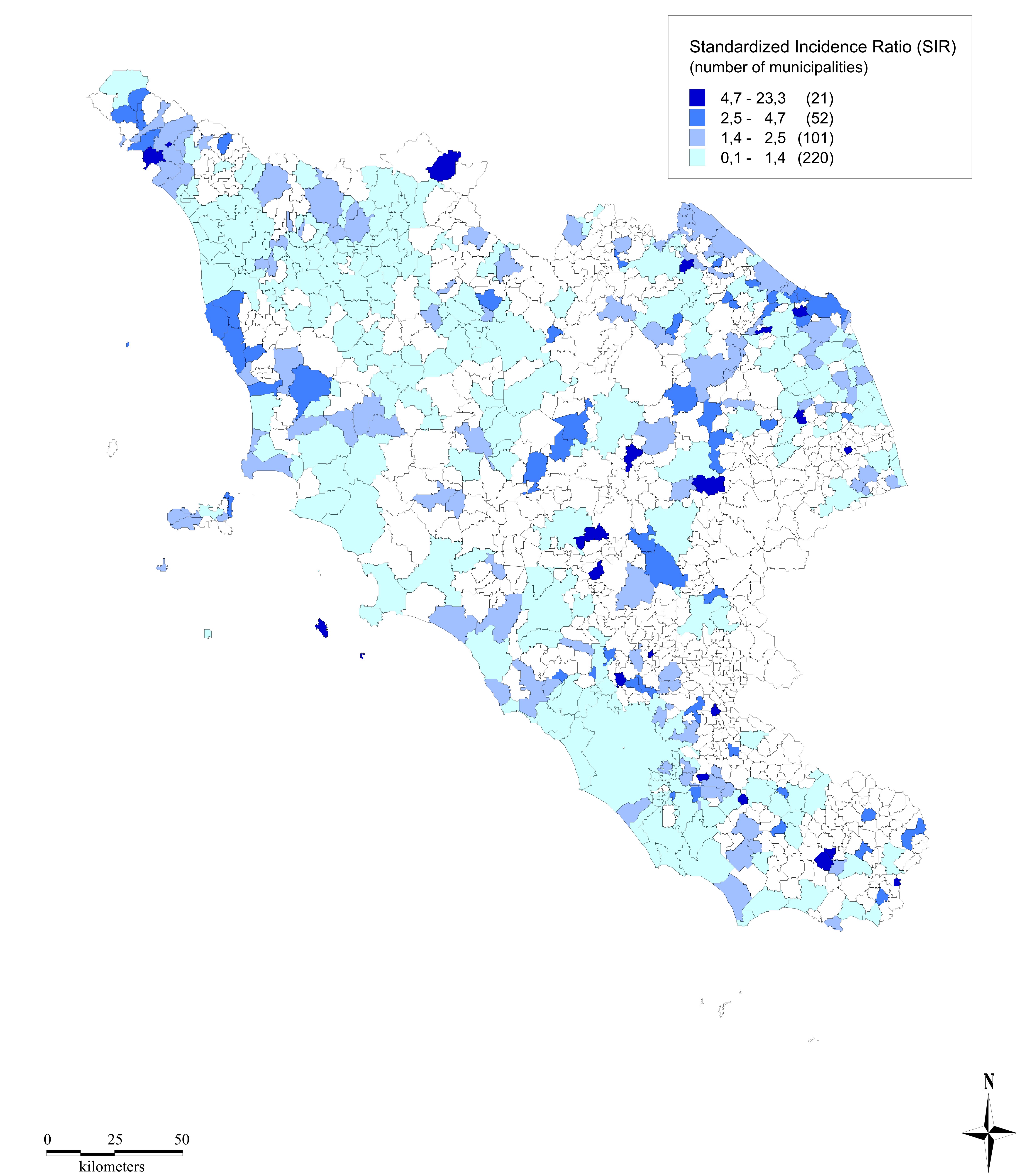

Supplement: Additional file 3: — Distribution of unadjusted standardized incidence ratio (SIR) of malignant mesothelioma in the Centre, Italy, ReNaM, 1993–2008. Crude SIRs of malignant mesothelioma (all sites) recorded by the Italian registry of malignant mesothelioma (ReNaM) in the 1993–2008 period are mapped based on municipality of residence. [file 12885_2015_1301_MOESM3_ESM.jpeg]

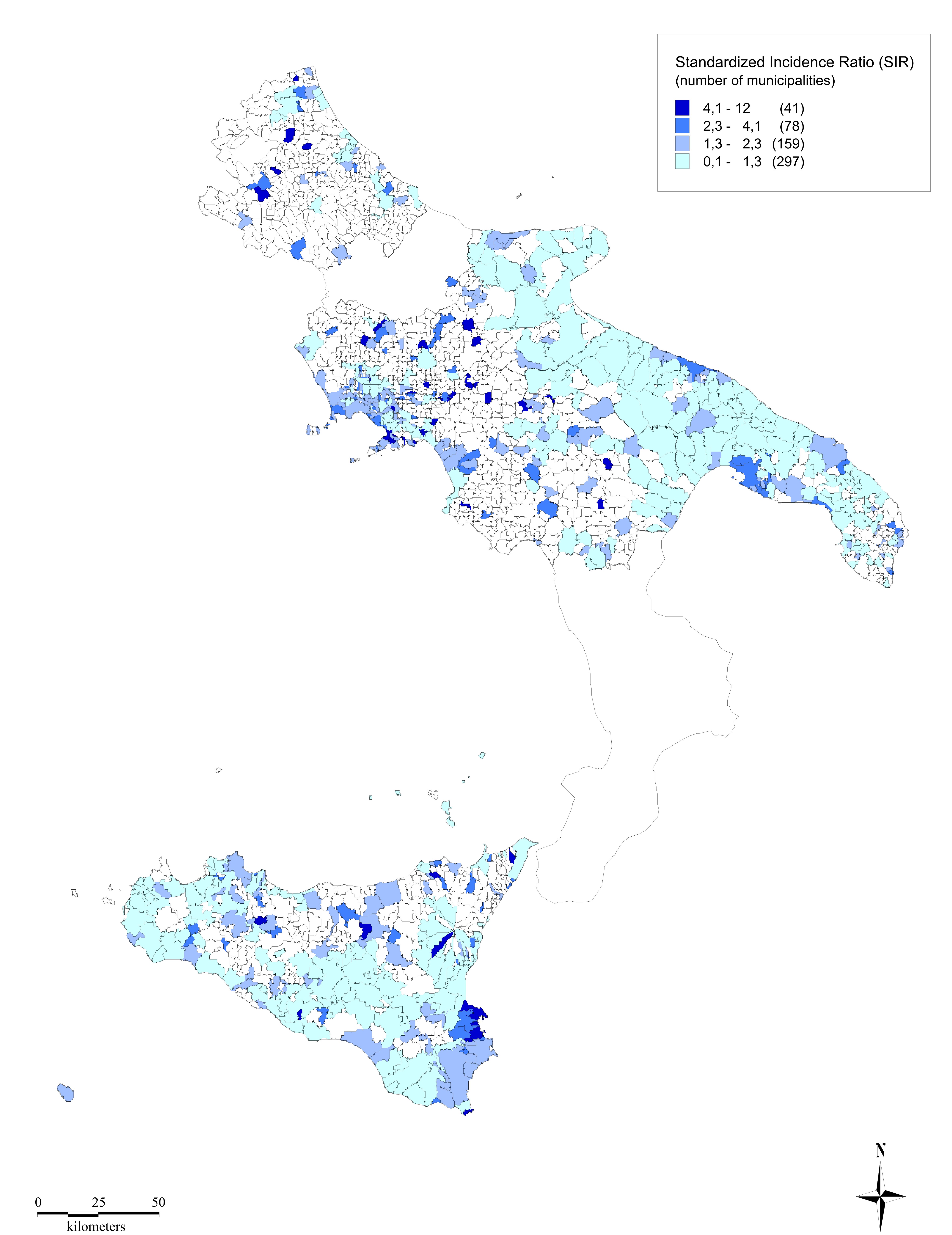

Supplement: Additional file 4: — Distribution of unadjusted standardized incidence ratio (SIR) of malignant mesothelioma in South & Islands of Italy, ReNaM, 1993–2008. Crude SIRs of malignant mesothelioma (all sites) recorded by the Italian registry of malignant mesothelioma (ReNaM) in the 1993–2008 period are mapped based on municipality of residence. No incidence data are available for Molise, Calabria and Sardinia (not shown). [file 12885_2015_1301_MOESM4_ESM.jpeg]

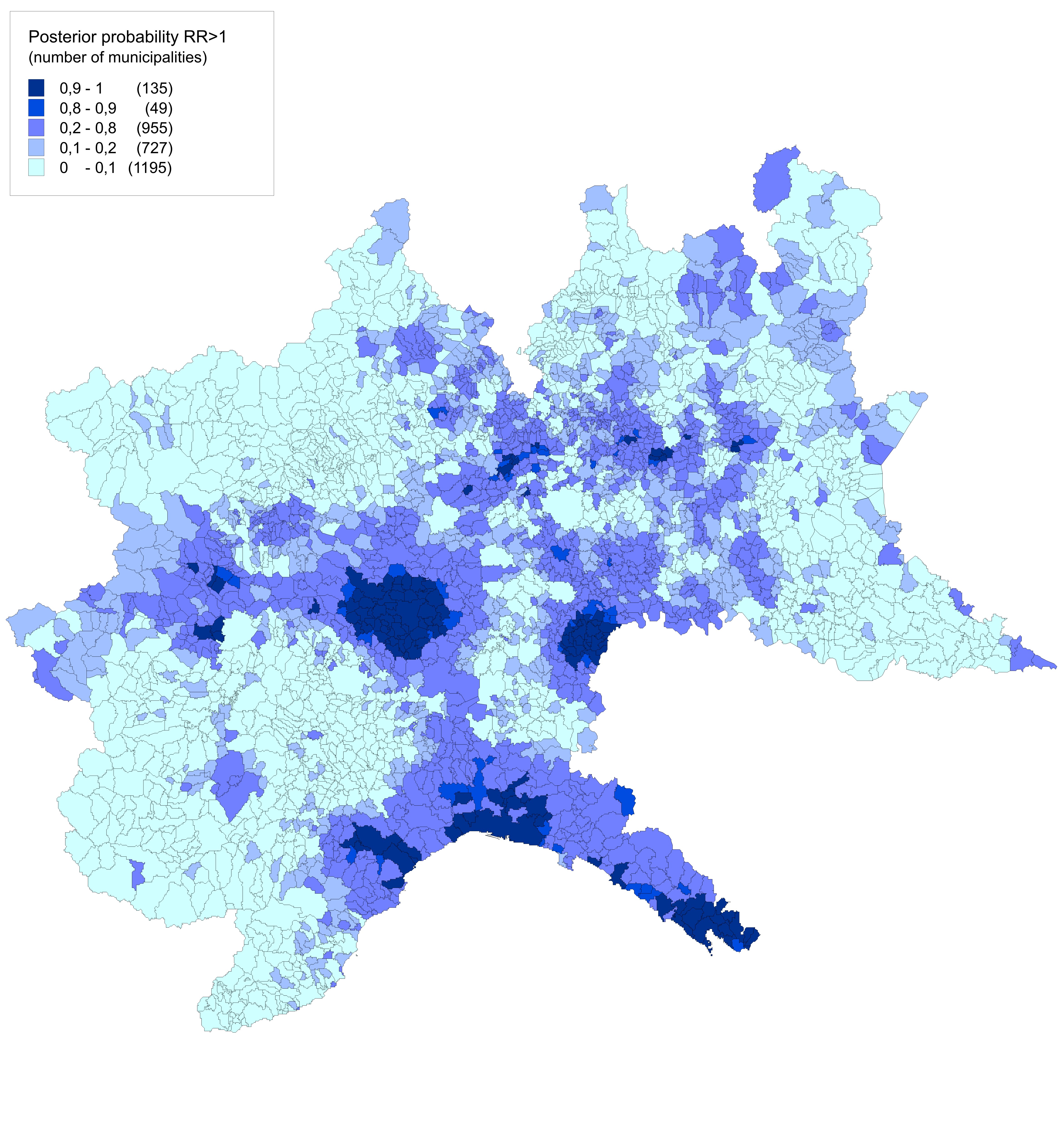

Supplement: Additional file 5: — Distribution of the posterior probability of smoothed relative risk (RR) being greater than 1 for malignant mesothelioma in the Northwest, Italy, ReNaM, 1993–2008. The mean posterior probability of RR >1 for malignant mesotheliomas (all sites) recorded by the Italian registry of malignant mesothelioma (ReNaM) in the 1993–2008 period is mapped based on municipality of residence. [file 12885_2015_1301_MOESM5_ESM.jpeg]

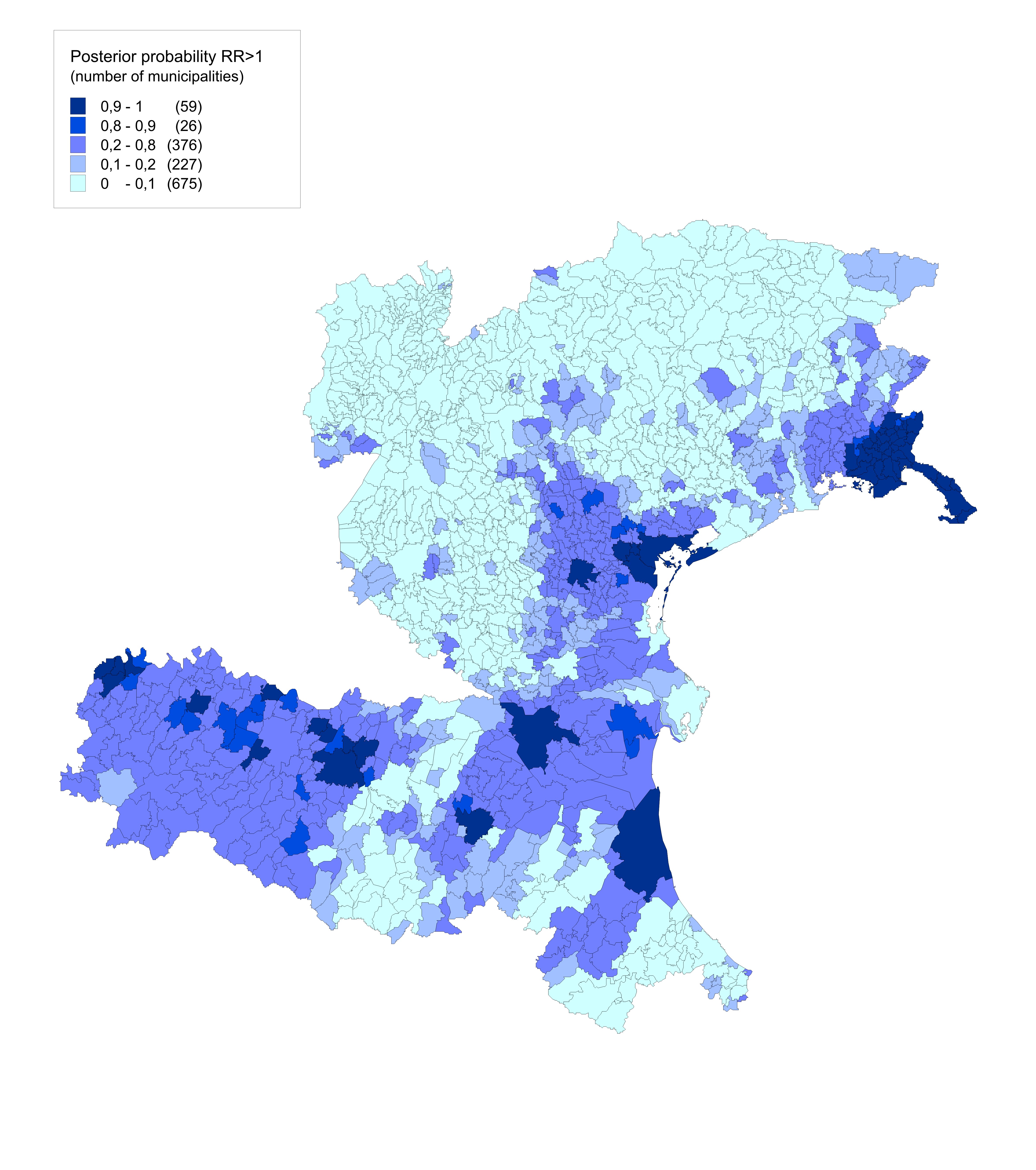

Supplement: Additional file 6: — Distribution of the posterior probability of smoothed relative risk (RR) being greater than 1 for malignant mesothelioma in the Northeast, Italy, ReNaM, 1993–2008. The mean posterior probability of RR >1 for malignant mesotheliomas (all sites) recorded by the Italian registry of malignant mesothelioma (ReNaM) in the 1993–2008 period is mapped based on municipality of residence. No incidence data are available for the autonomous province of Bolzano. [file 12885_2015_1301_MOESM6_ESM.jpeg]

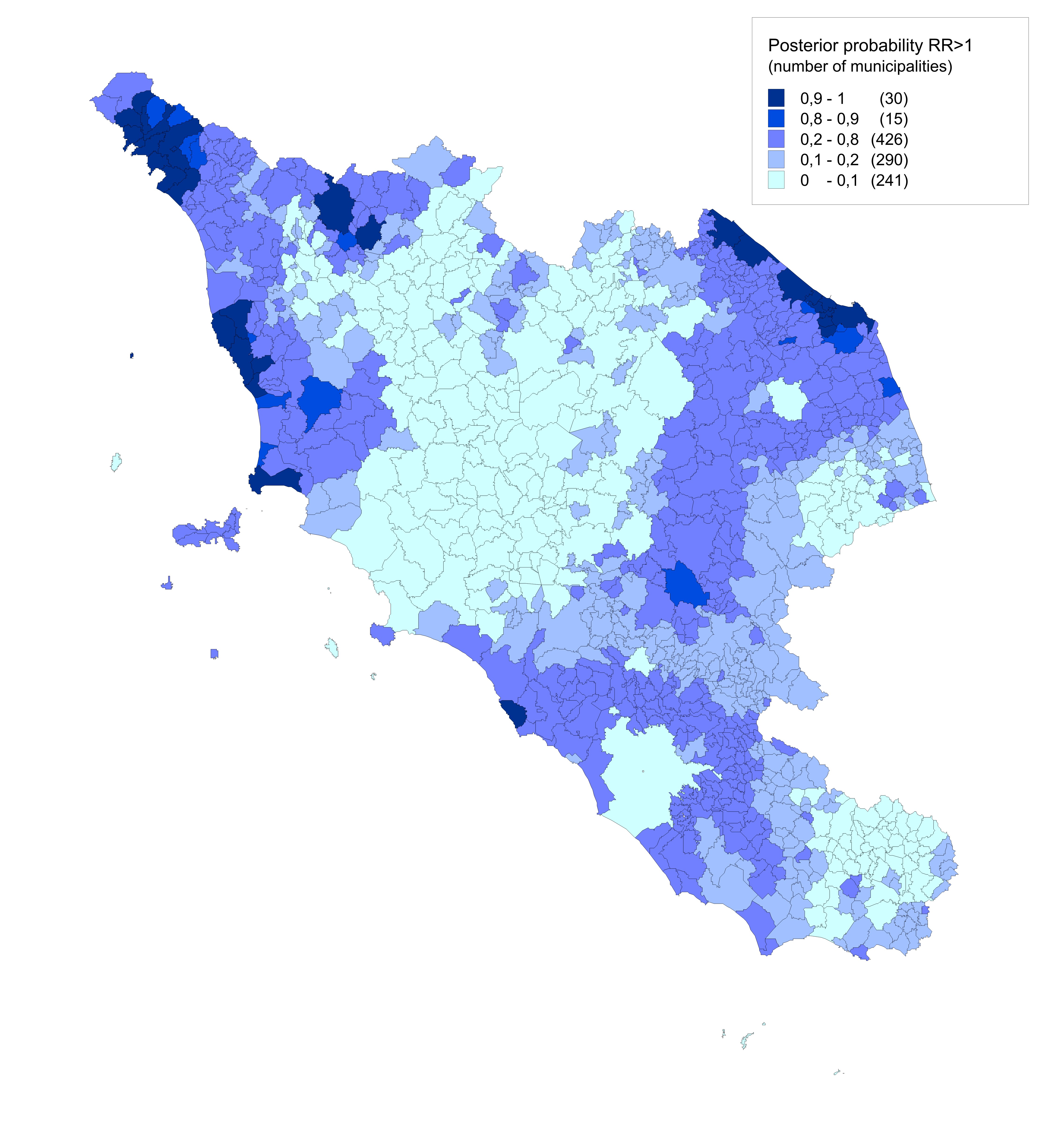

Supplement: Additional file 7: — Distribution of the posterior probability of smoothed relative risk (RR) being greater than 1 for malignant mesothelioma in the Centre, Italy, ReNaM, 1993–2008. The mean posterior probability of RR >1 for malignant mesotheliomas (all sites) recorded by the Italian registry of malignant mesothelioma (ReNaM) in the 1993–2008 period is mapped based on municipality of residence. [file 12885_2015_1301_MOESM7_ESM.jpeg]

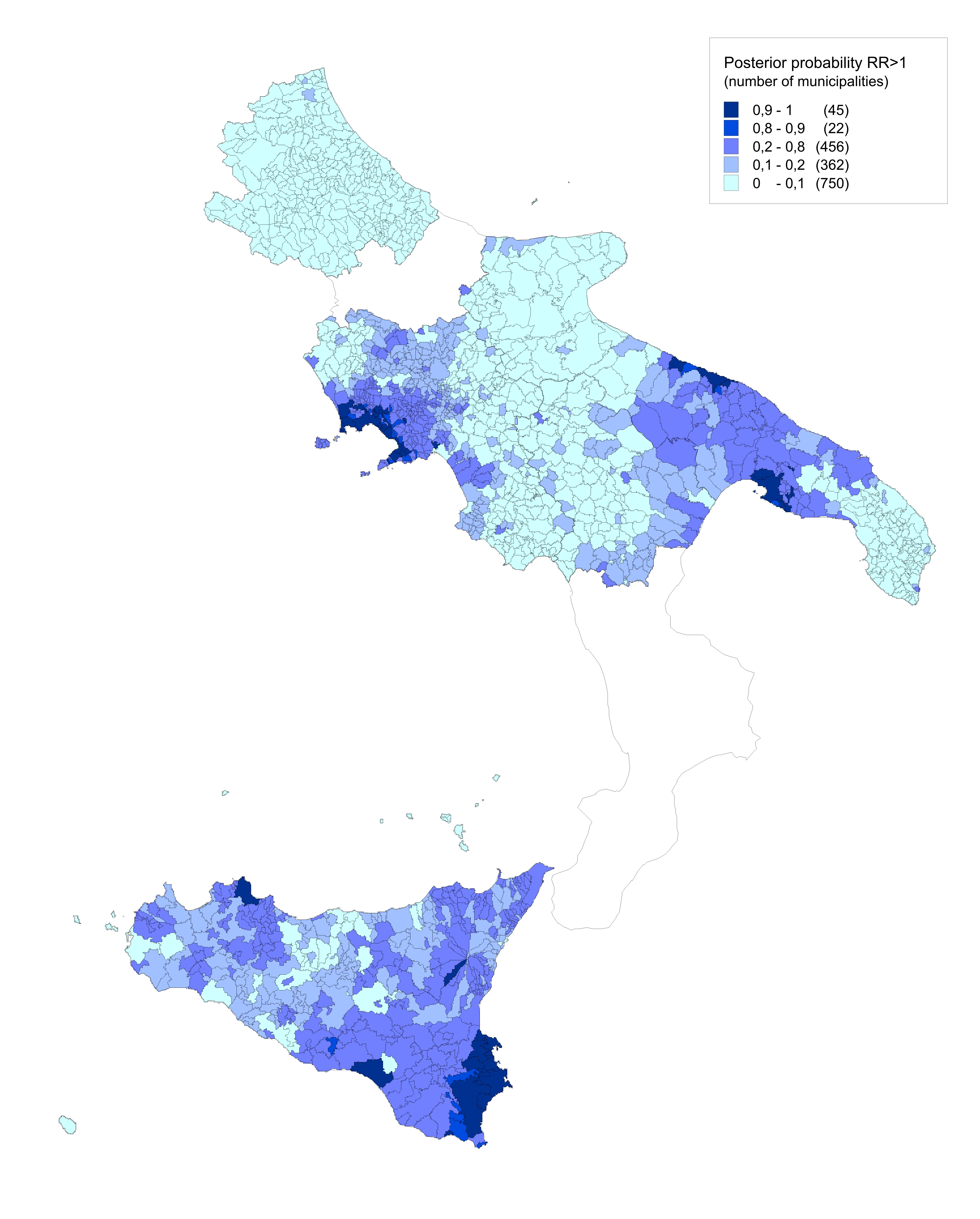

Supplement: Additional file 8: — Distribution of the posterior probability of smoothed relative risk (RR) being greater than 1 for malignant mesothelioma in South & Islands, Italy, ReNaM, 1993–2008. The mean posterior probability of RR >1 for malignant mesotheliomas (all sites) recorded by the Italian registry of malignant mesothelioma (ReNaM) in the 1993–2008 period is mapped based on municipality of residence. No incidence data are available for Molise, Calabria and Sardinia (not shown). [file 12885_2015_1301_MOESM8_ESM.jpeg]
